# Selective self-assembly of adenine-silver nanoparticles forms rings resembling the size of cells

**DOI:** 10.1038/srep17805

**Published:** 2015-12-08

**Authors:** Sungmoon Choi, Soonyoung Park, Seon-Ah Yang, Yujin Jeong, Junhua Yu

**Affiliations:** 1 Department of Chemistry Education, Seoul National University, 1 Gwanak-Ro, Gwanak-Gu, Seoul 151-742, South Korea

## Abstract

Self-assembly has played critical roles in the construction of functional nanomaterials. However, the structure of the macroscale multicomponent materials built by the self-assembly of nanoscale building blocks is hard to predict due to multiple intermolecular interactions of great complexity. Evaporation of solvents is usually an important approach to induce kinetically stable assemblies of building blocks with a large-scale specific arrangement. During such a deweting process, we tried to monitor the possible interactions between silver nanoparticles and nucleobases at a larger scale by epifluorescence microscopy, thanks to the doping of silver nanoparticles with luminescent silver nanodots. ssDNA oligomer-stabilized silver nanoparticles and adenine self-assemble to form ring-like compartments similar to the size of modern cells. However, the silver ions only dismantle the self-assembly of adenine. The rings are thermodynamically stable as the drying process only enrich the nanoparticles-nucleobase mixture to a concentration that activates the self-assembly. The permeable membrane-like edge of the ring is composed of adenine filaments glued together by silver nanoparticles. Interestingly, chemicals are partially confined and accumulated inside the ring, suggesting that this might be used as a microreactor to speed up chemical reactions during a dewetting process.

Constructing functional mesoscopic superstructures with nano-bricks is challenging and typically relies on the self-assembly of nanostructures as a bottom-up method^1^,^2^,^3^,^4^,^5^,^6^,^7^,^8^. Studies on the spontaneous association of components into a specific complex have dramatically advanced the development of functional nanomaterials, mostly at the nanoscale^9^,^10^,^11^,^12^,^13^,^14^. However, the structure of the macroscale multicomponent materials built by the self-assembly of nanoscale building blocks is hard to predict due to the multiple intermolecular interactions of great complexity^5^,^15^,^16^. A natural approach to the construction of the mesoscopic superstructure is to achieve a thermodynamically stable state by selected building blocks, for example, the utilization of amphiphilic polymer or biomacromolecules to build vesicles and filaments^17^,^18^. External forces such as electric field, bio-specific interactions and templates are also applied to direct the process of self-assembly^19^,^20^,^21^,^22^. Evaporation of solvents is another important approach to induce far-from-equilibrium effects, by which nanoparticles are trapped at a dynamic kinetic state and consequently a large-scale specific arrangement of building blocks is achieved^23^,^24^,^25^,^26^,^27^,^28^. Such a process in the natural drying/wetting cycles of surfaces due to rain, tidal or temperature fluctuations is also believed to allow the prebiotic synthesis and concentration of bioactive molecules^29^,^30^,^31^,^32^.

Metallic nanoparticles and nucleic acid have been used as building blocks for the nanoscale superstructure construction in which the hydrogen bonding pairs of the complementary sequences from two DNA molecules guide the assemblies of nanoparticles^14^,^33^,^34^,^35^. Silver nanoparticles have not been utilized as frequently as the gold nanoparticles in building metallic nanoparticle-DNA superstructures, likely due to the strong interaction between silver and nucleic acid that might interfere with the formation of well-organized structure^36^,^37^,^38^. Nevertheless, the nucleobase, adenine, forms supramolecular structures with some metal complexes^39^,^40^,^41^. We tried to monitor the possible interactions between nanoparticles and nucleic acid at a larger scale by epifluorescence microscopy. The similarity in ingredients between silver nanoparticles and luminescent silver nanodots enables the doping of silver nanoparticles with luminescent silver nanodots, and subsequently the actions of silver nanoparticles can be optically reflected by either a fluorometer or a fluorescence microscope^42^,^43^,^44^. Given the much lower cost of nucleobases compared to DNA sequences in the large-scale production of self-assembled superstructure, we investigated the interaction between silver nanoparticles and nucleobases in the concentration process of their aqueous solution via evaporation, and found that silver nanoparticles form ring-like superstructures resembling the size of cells.

## Results

**Figure 1:**
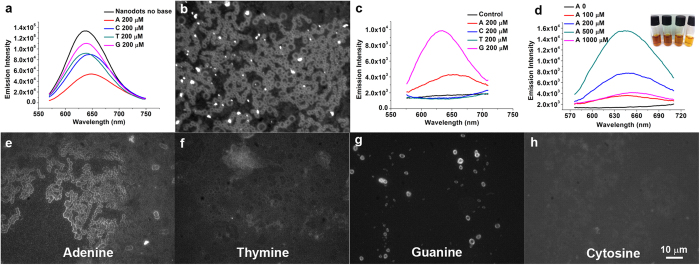
Detection of interactions between silver and nucleobases. (**a**) Emission spectra of C12-protected silver nanodots (10 μM C12:120 μM Ag) in the absence of any base (Nanodots no base) and in the presence of 200 μM adenine (A), cytosine (C), thymine (T) and guanine (G), respectively. (**b**) Fluorescence image of dewetted adenine and silver nanoparticles revealed by silver nanodots. Adenine (1 mM), silver nanoparticles (10 μM C12: 120 μM Ag) and C12-protected silver nanodots (5 μM C12: 30 μM Ag) were dried naturally at room temperature. Note that the bright emission from the aggregates of silver nanodots speckled the image. (**c**,**d**) *In situ* generation of silver nanodot-doped silver nanoparticles. (**c)** Among the four nucleobases, only guanine and adenine etched silver nanoparticles to generate luminescence silver nanodots. The “control” is the spectrum of silver nanoparticles in the absence of any base. (**d**) The emission intensity of silver nanodots depended on the concentration of adenine. (**e**–**h**), Fluorescence images of respective nucleobases and silver. Images were taken following the same procedure as in (**b**) except that chemicals were replaced with silver nanodots-doped silver nanoparticles (10 μM C12: 230 μM Ag) and a respective nucleobase (500 μM). The emission patterns were consistent with the photoluminescence spectroscopy in (**c**), in which adenine and guanine showed optically the interactions between purine and silver nanoparticles. Images having the same scale bar in (**h**).

Silver nanodots, which are protected clusters of silver atoms, show excellent brightness and photostability^42^,^43^,^45^. The size of the ssDNA-stabilized silver nanodots is similar to that of their protection group such as ssDNA, which is particularly useful for studying the interaction between nucleobases and ssDNA-stabilized silver nanoparticles as silver nanodots would be sterically invisible among the silver nanoparticles^46^. Another advantage of applying the silver nanodots in this study is their stability in the presence of nucleobases, as shown in Fig. 1a. Among the four nucleobases (adenine, thymine, cytosine and guanine, 200 μM), the emission of the 12-mer oligocytosine (C12)-protected silver nanodots retained more or less. The intensity shifts due to adenine and guanine might indicate possible interactions between the silver nanodots and the purine bases.

We subsequently examined the interaction between silver nanoparticles and adenine by drying a 20 μL aqueous solution of C12-stabilized silver nanoparticles (10 μM C12: 120 μM Ag), C12-protected silver nanodots (5 μM C12: 30 μM Ag) and adenine (1 mM) on a glass coverslip at room temperature. The fluorescence image of the dry sample under an epifluorescence microscope showed multiple rings tightly linked to form linear chains with diameters resembling the size of cells—a feature that has never been seen before (Fig. 1b). Regrettably, the image was speckled with much brighter spots that we ascribed to the emission of aggregated silver nanodots. To avoid these bright spots, we prepared silver nanoparticles that were homogeneously doped with silver nanodots by etching silver nanoparticles directly with nucleobases^44^. Again, the purines, but not pyrimidines, showed effects and led to the generation of luminescent silver nanodots (Fig. 1c). This was in line with the fluorescence microscope observation that only adenine/silver nanoparticles and guanine/nanoparticles samples showed fluorescence images after drying an aqueous solution of the silver nanoparticles (10 μM C12: 230 μM Ag) in the presence of a respective nucleobase (500 μM, Fig. 1e–h). Interestingly, the image pattern of ribbon/worm-like morphologies in Fig. 1e was obviously different from the previous (Fig. 1b). However, the image of the guanine showed bright distinct rings (Fig. 1g).

We then took the adenine as an example to explore the links between silver nanoparticles and the nucleobase. The optimal concentration of adenine for the generation of luminescent silver nanodots from silver nanoparticles was about 500 μM (Fig. 1d). At such a concentration, the dry sample of adenine alone showed bright oval rings when excited with near-UV band under microscope (Supplementary Fig. S1a). The ovals were also observed by scanning electron microscope (SEM, Supplementary Fig. S1b). The addition of C12 (10 μM) to adenine did not change its morphology (Supplementary Fig. S1c and S1d), but silver ions (450 μM) dispersed the ovals of adenine (Supplementary Fig. S1e), resulting in flakes as seen by SEM (Supplementary Fig. S1f). These aggregates were composed of adenine filaments that have been observed in the presence of metal derivatives (Supplementary Fig. S2)^41^. We did not detect any ovals rings from C12-stabilized silver nanoparticles or C12-etched C12-stabilized silver nanoparticles (Supplementary Fig. S1g – S1j)^44^. The control experiments indicated that the bright chains in Fig. 1b,e were determined by composite contributions from adenine and C12-stabilized silver nanoparticles.

**Figure 2:**
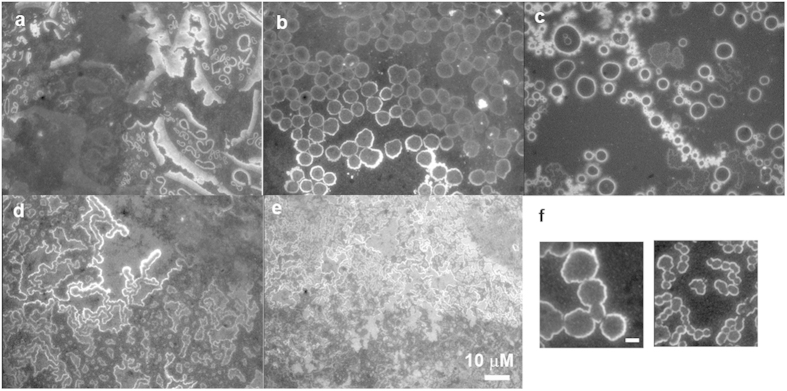
Dependence of mesoscopic adenine morphology on silver contents. Fluorescence images showed the transformation of dewetted adenine (500 μM) from fragments of rings to rings, to ribbons and then to indiscernible structures when the silver content was increased. The concentrations of silver from (**a**–**g)** were 85 μM, 125 μM, 185 μM, 230 μM and 280 μM, respectively, while keeping the same concentration of C12 (10 μM). (**f)** Close-up images showing the merging of adjacent rings. Images having the same scale bar in (**e)** except (**f)** that is also 10 μm.

**Figure 3:**
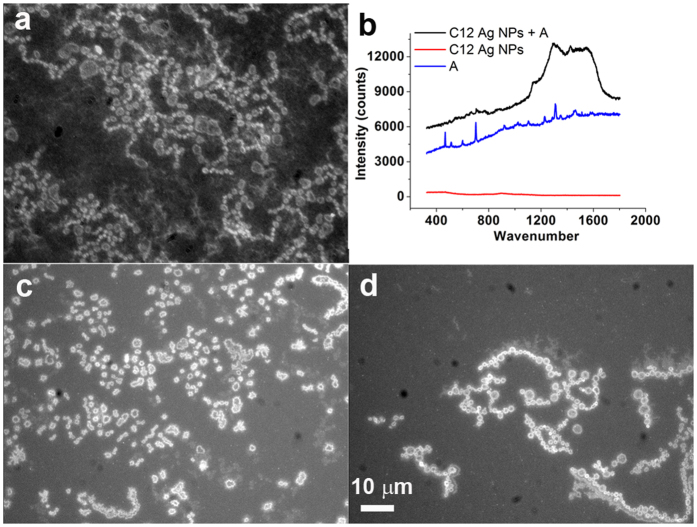
Typical morphology of adenine in the presence of silver nanoparticles. (**a**) The dewetted adenine (500 μM) in the presence of silver nanodot-doped silver nanoparticles showed tightly contacted bright rings, which appeared to be curved chains. Such patterns were observed at all the examined silver contents. (**b**) Raman spectroscopy of adenine and silver. Raman peaks from adenine (blue line) were also found from the adenine-silver nanoparticle sample (black line) but not from the silver nanoparticle sample (red line). However, the large emission peak in the black line indicated that the photon source of the fluorescence images were from the photoluminescence of silver nanodots that were doped homogeneously into silver nanoparticles. (**c**,**d**) Same as (**a**) except that the samples were diluted 10-fold (**c**) and 100-fold (**d**). Images having the same scale bar in (**d**).

The fluorescence patterns in the images depended on the silver content in the mixture of adenine/silver nanoparticles. The addition of the silver nanoparticles dismantled the ovals of adenine. Subsequently, bright fragments of rings, rings, linked rings (chains) and merged rings (worm/ribbons) appeared in the fluorescence images. The most indiscernible structures at a low silver content (85 μM Ag) were the fragments of rings with wide bright edges ranging from 0.3 μm to 4 μm (Fig. 2a). At a higher silver nanoparticles concentration (125 μM Ag), all the fragmented rings evolved into rings that contacted their neighbours (Fig. 2b) with a wide size distribution centred at 5.5 μm (Supplementary Fig. S3a). These rings were obviously larger and not circular compared to the typical rings/chains that appeared at all silver concentrations with a narrower size distribution centred at 1.5 μm (Fig. 3a, Supplementary Fig. S3b). A further increased silver content (185 μM Ag) resulted in smoothed, smaller rings (Fig. 2c, Supplementary Fig. S3c). Note that some of the contacted rings started to merge into a single compartment as shown in Fig. 2f. The merging became dominant and only merged rings (worm/ribbons) were observed when silver content was too high (230 μM Ag, Fig. 3d). No discernible structures were obtained as silver reached 280 μM (Fig. 2e).

**Figure 4:**
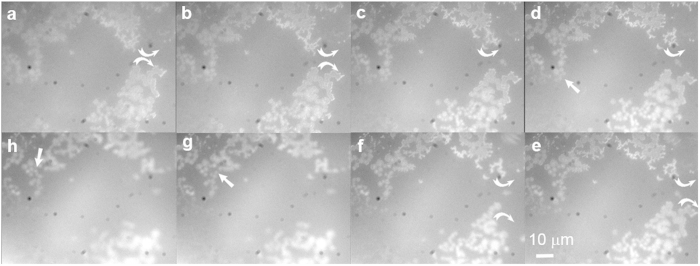
Self-assembly of adenine and silver nanoparticles. Screenshots of a video clip taken during the dewetting of adenine (500 μM) in the presence of silver nanodot-doped silver nanoparticles (10 μM C12: 180 μM Ag, Supplementary Information). These images showed the move and rearrangement of rings and ribbons, as partially marked by arrows, suggesting that adenine and silver nanoparticles had self-assembled before fully dewetted. (**a**–**h**) were taken in chronological order. Images having the same scale bar in (**e**).

The typical morphology of the dry sample of adenine and silver nanoparticles in the fluorescence images was the rings/chains structure (Fig. 3a). However, it was hard to distinguish the rings/chains using a bright-field image of the sample (Supplementary Fig. S4). The size of the rings mainly ranged from 1 micron to 3 microns (Supplementary Fig. S3b). Rings were either distinct or contacted their neighbours tightly to form curved chains. We confirmed by Raman spectroscopy that the luminescence but not the light scattering from silver nanoparticles appeared in the fluorescence images (Fig. 3b). The brighter-than-the-background internals of the rings indicated that the silver nanoparticles were concentrated in the rings and the majority accumulated at the edges of rings. Interestingly, when the concentration of the above mixture was 10-fold lower (Fig. 3c) or even 100-fold lower (Fig. 3d) we were still able to observe the rings/chains structure with little change in their size (Supplementary Fig. S5). The major difference of spontaneous self-assembly from dewetting-induced aggregation is that the self-assembly can occur both in solution and on the surface, whereas the latter only happens on the surface. Silver nanodots doped nanoparticles were especially useful for long-term optical imaging in solution thanks to their much better photostability than organic dyes and silver nanodots (Supplementary Fig. S6). We thus pointed the focus point towards the liquid phase and observed the self-assembly of adenine/nanoparticles in aqueous solution (Fig. 4 and Supplementary video), in which rings and chains moved and rearranged as indicated by arrows in Fig. 4. This strongly supported the idea that rings had formed and assembled further to chains before the sample became dry.

The self-assembly was reasonably stable. The bright rings retained in the presence of 3 mM polyethylene glycol (PEG) in the above solution but the chains were dismantled, likely because the surfactant weakened the inter-ring interactions (Supplementary Fig. S7). Similarly, these rings were partly kept in the presence of glycine, a simple amino acid, up to 1 mM in the above solution (Supplementary Fig. S8a and S8b). However, the structure of the self-assemblies of the silver nanoparticles and adenine could be changed. A model protein, avidin, broadened the ring, indicating that such rings might be able to survive in a certain complicated environment (Supplementary Fig. S8c). When gold ions were added to the solution of silver nanoparticles and adenine, the emission was quenched and we could not observe any pattern from the fluorescence images. Copper did not quench the emission in a similar manner as the gold, but no ring was observed from the fluorescence images as well as SEM images (Supplementary Fig. S9).

**Figure 5:**
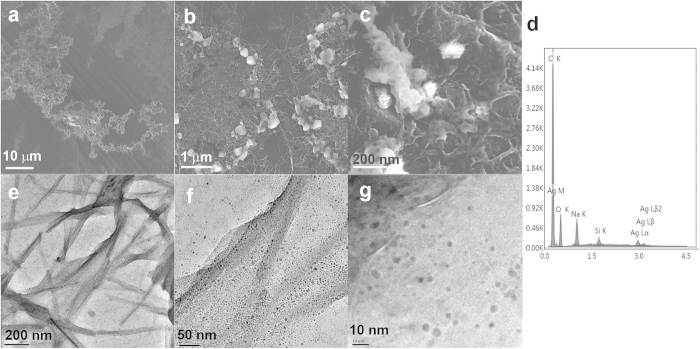
Characterization of adenine-silver nanoparticle self-assembly by electron microscopy. (**a**–**c**) SEM images at various magnifications showing the disc-like assembly of adenine and silver as well as the accumulation of amorphous nanoparticles. (**d**) EDS analysis suggesting that the previous amorphous particles were organic compounds and silver hybrids. Such a phenomenon implied that the edges of the discs of the adenine-silver nanoparticle self-assembly formed a virtual boundary. (**e**–**g**) TEM images at various magnifications showing that numerous silver nanoparticles adsorbed on the filaments of adenine. Silver nanoparticles likely acted as glue to link adenine filaments together to form disc-like mesoscopic architectures.

**Figure 6:**
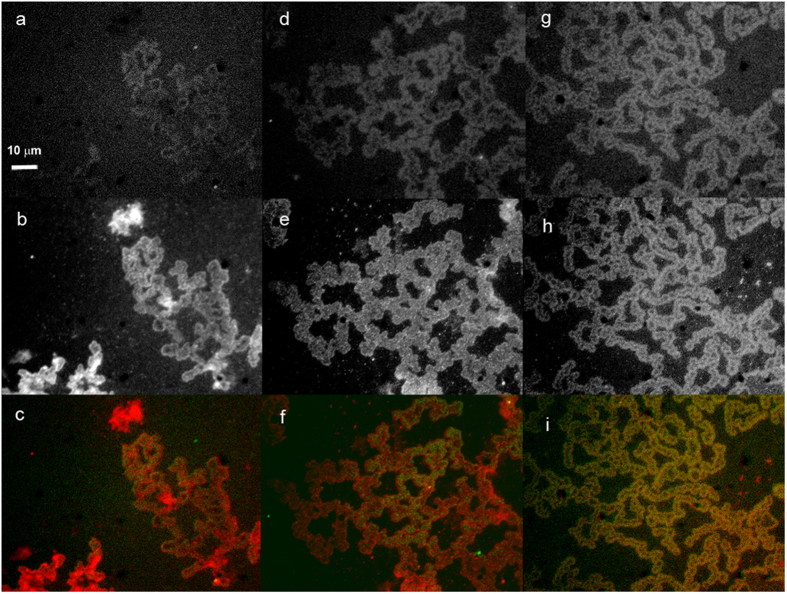
Chemical reaction inside the rings of adenine and silver nanoparticles. Aqueous solution of adenine (500 μM), silver-nanodot-doped silver nanoparticles, glycine and ninhydrin was dropped on a glass coverslip and imaged under UV light excitation (the first row) or green light excitation (the second row). In the colocalization image (the third row), pseudocolor red was for silver nanodots emission and pseudocolor green for emission from the product of glycine and ninhydrin reaction. The concentrations of glycine and ninhydrin in the columns (from left to right) were 50 μM, 150 μM and 250 μM, respectively. It shows that the emission intensity of the above reaction product was higher inside the rings, suggesting that the ring of adenine/nanoparticles might have encircled the glycine-ninhydrin reaction pair to facilitate the reaction. Images having the same scale bar in (**a**).

SEM images of the self-assembly of silver nanoparticles and adenine showed disc-like hybrids in which filaments were bundled into larger interconnected architectures of several micrometers in diameter (Fig. 5a–c). The detailed structure obtained by TEM demonstrated the adsorption of silver nanoparticles on the filament of adenine (Fig. 5d–f) with the majority of silver nanoparticles less than 5 nm (Supplementary Fig. S10). The driving force of the self-assembly might be ascribed to the strong interaction between silver nanoparticles and adenine filaments, in which silver nanoparticles glued adenine filaments together. The edge of the discs was embedded with amorphous nanoparticles of organic and silver hybrids as revealed by EDS analysis (Fig. 5b,d). The embedded nanoparticles and residues at the edges were likely due to capillary flow of nanoparticles and salts along the filaments and the accumulation at the edge^47^. This implied that the frame of the self-assembly virtually formed a boundary and molecules would be confined inside it. Such a scenario was in line with the fluorescence images in which the internals of the rings/chains were brighter than the background. Such an enrichment might significantly increase the local concentration of species, resulting in accelerated reaction rates of confined molecules^48^. We indeed observed more products accumulated inside the rings when evaporating the silver nanoparticles-adenine solution in the presence of a reaction pair, glycine and ninhydrin, that produces emissive species^49^. We checked the lowest concentration of glycine and ninhydrin to be 50 μM at which we could observe the emission image from the product of glycine and ninhydrin reaction in the presence of the ring structures (Fig. 6a). The higher the concentration of glycine and ninhydrin, the brighter the image from the emission of the above product (Fig. 6a,d,g; Supplementary Fig. S11). The emission patterns from the product of glycine and ninhydrin colocalized well with the emission patterns of silver nanodots in the superstructure of silver nanoparticles and adenine (Fig. 6a–i). However, no emission was observed from the images of glycine and ninhydrin in the absence of the ring structure. When we further increased the concentration of glycine and ninhydrin to 50 mM, we observed emission images with a similar intensity to that in the presence of the ring structure. Therefore, it is a roughly 200-fold increase in the reaction possibility.

## Discussion

Generally, the rupture of a film occurs at locations with a low density of nanoparticles during the dewetting of the liquid film^50^. Depending on the coverage of the nanoparticles, the morphology of aggregated nanoparticles can be islands, ribbons or networks^24^. However, in our study, a similar concentration of silver nanoparticles resulted in morphology variations as observed by fluorescence imaging (Fig. 1), suggesting that the interaction between nucleobases and nanoparticles but not the dewetting process played a major role in determining the fluorescence morphology. Moreover, the characteristic ring structure in the mixture of silver nanoparticles and adenine retained even after a 100-fold dilution (Fig. 3). This was opposite to the discovery that a lower nanoparticle coverage leads to isolated islands^24^. Therefore, self-assembly might play an important role in the formation of patterns in the fluorescence images. This was further confirmed by the observation of the self-assembly in solution.

Silver ions show strong affinity for nucleobases^36^. It did not appear that silver nanoparticles and adenine were linked mainly by electrostatic force as silver nanoparticles were neutral with a zeta potential of −7 mV. It is quite likely that the residual silver ions at the surface of the nanoparticles interacted with the nucleobase to stabilize the superstructure of silver nanoparticles and adenine filaments. It has been reported that silver ions and adenine form supramolecular structure^39^. However, reduced silver can be stabilized with ssDNA due to the strong interaction between the nucleobase and the reduced silver^51^,^52^. It is hard to distinguish which interaction, the reduced silver-adenine or the silver ion-adenine, is the main force to “glue” the silver nanoparticles and adenine filaments together. The morphology of the superstructure of the silver nanoparticles-adenine assembly depended on the interactions between silver nanoparticles and adenine. The addition of external molecules may disturb the above interactions including the change-induced destabilization, inducing the structural change of the superstructure.

The high reaction possibility of a reaction pair inside the rings indicated that the self-assembly of adenine and silver nanoparticles might have partially acted as membranes to confine molecules for bio-oriented reactions^53^. The bright emission in the fluorescent images indicated the location of silver nanoparticles/nanodots. Shown both in the fluorescence images and EM images, there were more filaments and silver nanoparticles/nanodots at the edge of the rings. Since the rings consist of filament-nanoparticle networks, the frame of the silver nanoparticles-adenine assembly should be permeable in solution. It acted partially as a permeable membrane only when the solution of the assembly underwent dewetting, in which molecules were trapped in the network of the frame, as shown in the SEM images in Figs 5 and 6.

In summary, luminescent silver nanodots revealed the self-assembly of silver nanoparticles and adenine filaments to form ring-like architectures of a size similar to modern cells. The higher silver content leaded to the merging of rings, yielding worm-like structures. The nanoparticles and residues accumulated near the edge of rings during the drying process, suggesting that the rings acted virtually as permeable membranes. The permeable membrane-like edge of the ring is composed of adenine filaments glued together by silver nanoparticles. The concentration of adenine/silver inside the virtual membranes may enrich the molecules and potentially accelerate the reactions among the confined molecules during a dewetting process.

## Methods

### Materials

Silver nitrate (anhydrous, 99.999%), sodium borohydride (powder, ≥98.0%), adenine, guanine, thymine, cytosine, sodium hydroxide and poly(ethylene glycol) (average M_n_ 3,350, powder), avidin, rhodamine B, ninhydrin and glycine were purchased from Aldrich and used without further purification. 12mer polycytosine was obtained from Integrated DNA Technologies. Fisherfinest™ Premium Cover Glasses were purchased from Fisher Scientific and used without further treatment. TEM grids (Carbon Grid Type-A, 300Mesh, Cu) were purchased from TedPella. Ultrapure (Nanopure system) filtered water with a resistivity 18.2 MΩ cm was used in all experiments.

### Instruments

HRTEM images were obtained on JEM 3010 high resolution transmission electron microscope. Field-Emission Scanning Electron Microscope (Carl Zeiss, SUPRA 55VP) was used to obtain SEM images and EDS analysis. Emission spectra were obtained on QM-40 (Photon Technology International, Inc.). A micro-Raman system (JY-Horiba, LabRam 300) was utilized for Raman spectrum recording with the 514.5 nm laser line from a Ar ion laser (Melles Griot, 35-MAP-321) for Raman excitation. Fluorescence images were obtained on Olympus X81 microscope coupled to an Andor Luca^EM^S 658 M camera. Zeta potential was measured on NanoPlus-1 (Particulate Systems).

### Preparation of stock solutions

i. Silver ion stock solution (14 mM). AgNO_3_ (12 mg) was dissolved in DI water (5 mL).

ii. Sodium borohydride stock solution (1 mg/mL). Sodium borohydride (3 mg) was dissolved in DI water (3 mL).

iii. Nucleobase stock solution (50 mM). Take adenine for example. Adenine (13.5 mg) was dissolved in an aqueous solution of sodium hydroxide (0.1 M, 2 mL).

iv. ssDNA stock solution (1 nmole/μL). ssDNA (650 nmole) was dissolved in DI water (650 μL).

### Synthesis of silver nanoparticles

Depending on the DNA/silver ratio, C12 stock solution (40 nmol, 40 μL) and corresponding AgNO_3_ stock solution were mixed in DI water (930 μL) and left it in dark for 1.5~2 hrs, followed by reducing with fresh sodium borohydride stock solution (37 μL). The sample was used after an overnight incubation in dark. The sample was then diluted in DI water (3 mL).

### Synthesis of silver nanodots

C12 stock solution (40 nmol, 40 μL) and AgNO_3_ stock solution (240 nmol, 17 μL) were mixed in DI water (950 μL) and left it in dark for 1.5~2 hrs, followed by reducing with fresh sodium borohydride stock solution (17 μL). The sample was used after an overnight incubation in the dark.

### Dewetting of silver nanoparticle/nucleobase

Corresponding C12-stabilized silver nanoparticles and nucleobases were mixed in DI water. 20 μL of the above solution was dropped onto a glass coverslip and left in the dark at room temperature. It was then imaged under an Olympus X81 microscope before or after being fully dewetted. Samples were excited with 100 W mercury lamp filtered by a 545–580 nm band pass filter and monitored after filtered by a 610 nm long pass filter (green excitation). The filter sets for adenine (UV excitation) were BP 360–370 for excitation and BP 420–460 for emission. For SEM and TEM imaging, samples were dropped on TEM grids.

### Reaction between glycine and ninhydrin

Glycine (10 μL, 100 mM) and ninhydrin (10 μL, 100 mM) were mixed and dropped onto a glass coverslip. The solution was left dry naturally and imaged with an epifluorescence microscope with a filter setting of BP 360–370 for excitation and BA 510–550 for emission. Lower concentrations of glycine and ninhydrin were diluted accordingly from the 100 mM stock solutions and mixed with corresponding silver nanoparticles and adenine.

## Additional Information

**How to cite this article**: Choi, S. *et al.* Selective self-assembly of adenine-silver nanoparticles forms rings resembling the size of cells. *Sci. Rep.* **5**, 17805; doi: 10.1038/srep17805 (2015).

## Supplementary Material

Supplementary Information

Supplementary video
