# Supplementary material for: Selective self-assembly of adenine-silver nanoparticles forms rings resembling the size of cells
Source: Sci Rep. 2015 Dec 8;5:17805. doi: 10.1038/srep17805 (PMC4672301; doi:10.1038/srep17805)
Supplement: Supplementary Information [file srep17805-s1.pdf]

Selective self-assembly of adenine-silver nanoparticles forms rings resembling the size of cells

Sungmoon Choi, Soonyoung Park, Seon-Ah Yang, Yujin Jeong and Junhua Yu*

Department of Chemistry Education, Seoul National University, 1 Gwanak-Ro, Gwanak-Gu, Seoul 151-742, South Korea

Correspondence to: Junhua Yu (Email: junhua@snu.ac.kr)

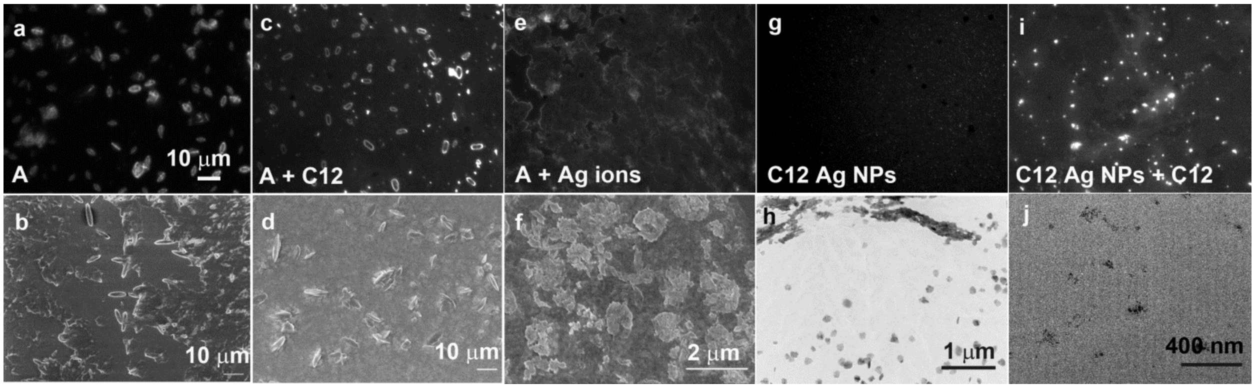

Fig. S1. **Interactions between silver and adenine.** **a**,**c**,**e**,**g**,**i**, Fluorescence image of dewetted adenine (500 μM, **a**), adenine (500 μM) and C12 (10 μM, **c**), adenine (500 μM) and Ag^+ (450 μM, **e**), C12-stabilized silver nanoparticles (10 μM C12 and 120 μM Ag, **g**), C12-stabilized silver nanoparticles and extra C12 (10 μM, **i**). Samples **a**, **c** and **e** were excited with light filtered by a BP 360-370 filter and monitored after being filtered by a BP 420-460 filter. Samples **g** and **i** were excited with light filtered by a BP 545-580 filter and monitored after being filtered by a LP 610 filter. **b**,**d**,**f**, SEM images of the corresponding samples on the first row. **h**,**j**, TEM images of sample **g** and **i**. The top row images share the same scale bar as the one in **a**.

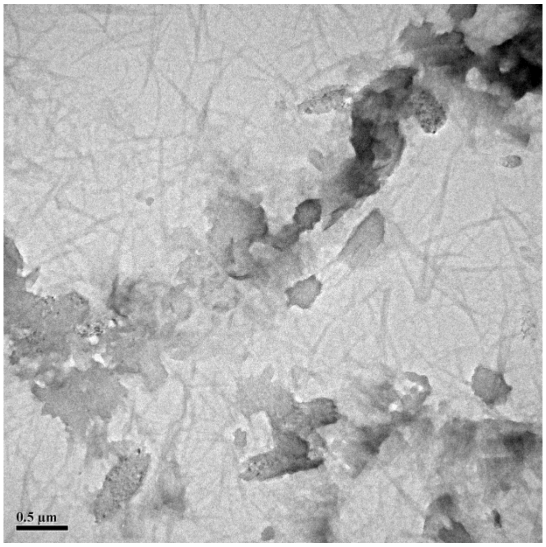

Fig. S2. **SEM image of adenine**. Filaments were observed in the absence of metal ions. It depended on the pH of the solution. Adenine has higher solubility in basic aqueous solution and it was hard to obtain ovals of adenine at pH 13.

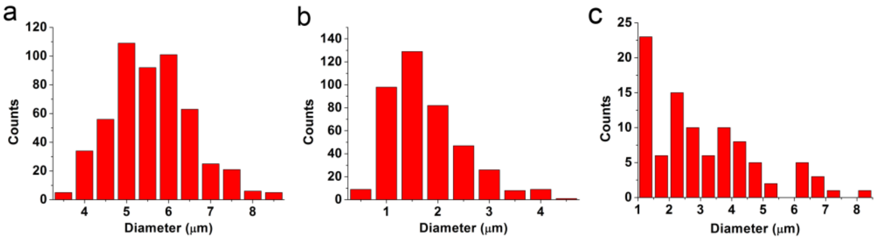

Fig. S3. **Size distribution of the self-assemblies of silver nanoparticles and adenine**. Ring size distribution at a silver nanoparticles concentration (125 μM Ag, **a**); typical ring size of the assembly of silver nanoparticles and adenine (185 μM Ag, **b**) and the size distribution at areas showing larger rings (**c**).

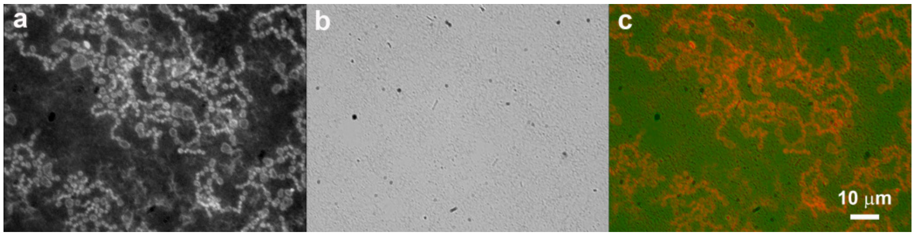

Fig. S4. **Colocalization between fluorescence and bright field images of adenine and silver nanoparticles**. Fluorescence image (**a**) and bright field image (**b**) of adenine and silver nanoparticles. In the colocalization image, pseudocolor red was for silver nanodots emission and pseudocolor green for bright field image.

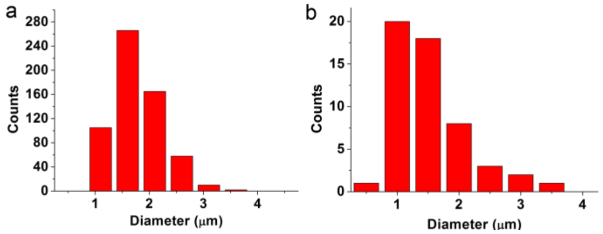

Fig. S5. **Size distribution of the self-assemblies of silver nanoparticles and adenine**. 10-fold (**a**) and 100-fold (**b**) dilution of the regular solution.

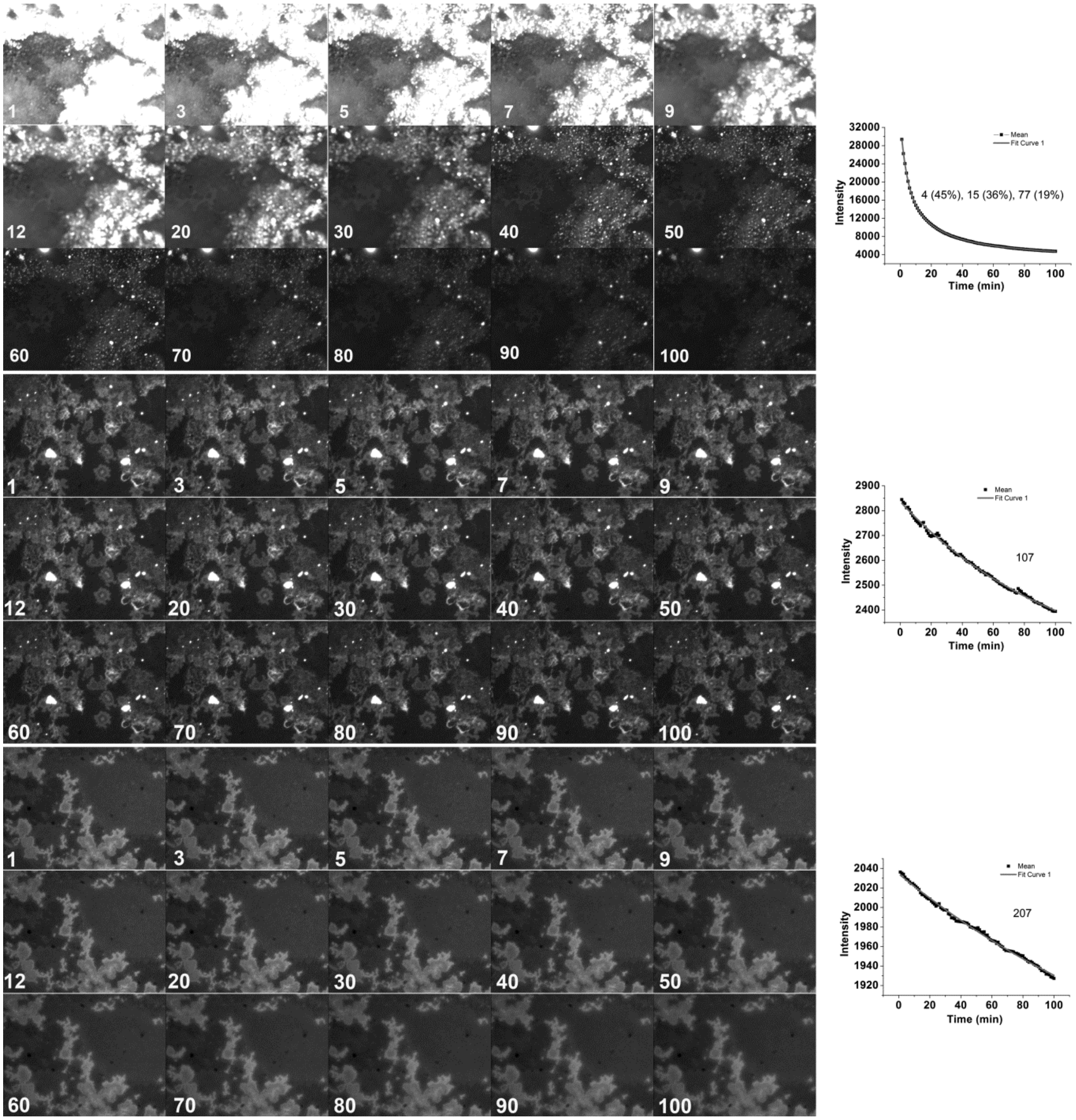

Fig. S6. **Comparison of photostability**. Video clips of samples taken under irradiation of green light. Videoshots for rhodamine B (top), silver nanodots (middle) and silver nanodots-doped nanoparticles (bottom) were shown at the given time. The right figures show the intensity decay of corresponding fluorophores, in which the numbers were the photostability lifetime obtained by monoexponential fitting the decay curve.

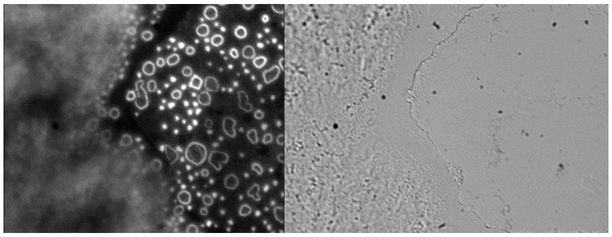

Fig. S7. **Adenine and silver nanoparticles in the presence of PEG**. Fluorescence image (left) and bright field image (right) show that the typical ring structures were kept in the presence of PEG (3 mM). The thick layer of PEG blocked the emission of nanodots on the left region of the images.

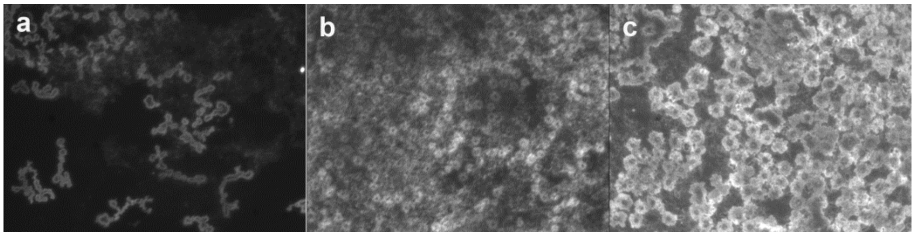

Fig. S8. **Adenine and silver nanoparticles in the presence of glycine and avidin**. **a**,**b**, Fluorescence images of adenine and silver nanodot-doped silver nanoparticles in the presence of 10 μM (**a**) and 1 mM (**b**) of glycine, or avidin (20 ng/mL, **c**).

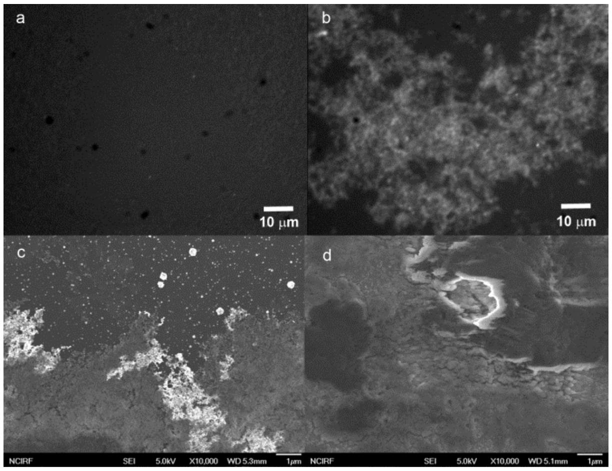

Fig. S9. **Interactions between adenine and gold or copper**. **a**,**b**, Fluorescence images of adenine and silver nanoparticles in the presence of gold (**a**) or copper (**b**). **c**,**d**, SEM images of adenine in the presence of gold (**c**) or copper (**d**) nanoparticles. The gold and copper nanoparticles were prepared following the protocol for silver, in which C12 and metal ions were mixed followed by sodium borohydride reduction.

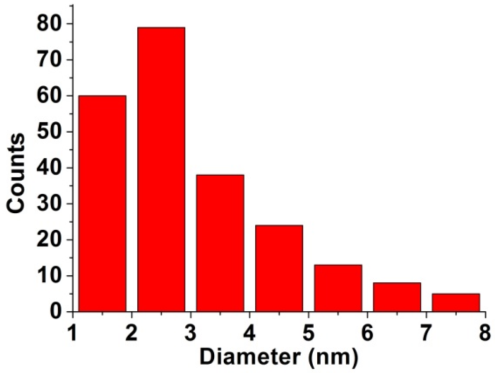

Fig. S10. **Size distribution of silver nanoparticles in the self-assembly of silver nanoparticles and adenine.**

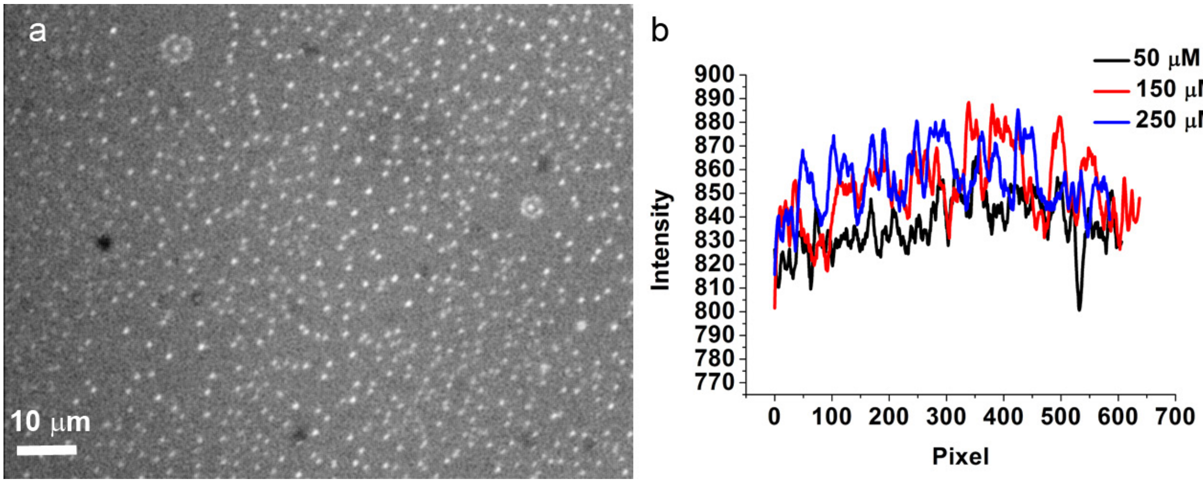

Fig. S11. **Raction between glycine and ninhydrin**. **a**, Fluorescence images of glycine and ninhydrin (25 mM) in the absence of silver nanoparticles and adenine. **b**, Plots of the emission intensity in Figure 6a, 6d and 6g, respectively. A line was drawn across the image and the intensity was plotted.

Supplementary video: Self-assembly of silver nanoparticles and adenine in aqueous solution. Video was recorded during the evaporation of solvents in an aqueous solution of silver nanoparticles and adenine. Ring-like structures ascribed to the self-assembly of silver nanoparticles and adenine were observed before the solution became dry.
